# Eggshell membrane as promising supplement to maintain bone health: A systematic review

**DOI:** 10.1016/j.bonr.2024.101776

**Published:** 2024-05-24

**Authors:** Johannes-Paul Fladerer, Selina Grollitsch

**Affiliations:** a Institute of Pharmaceutical Sciences, University of Graz, Beethovenstraße 8, 8010 Graz, Austria; b Apomedica Pharmazeutische Produkte GmbH, Roseggerkai 3, 8010 Graz, Austria

**Keywords:** Bone density, Glycosaminoglycans, Collagen type I, Bone mineralization

## Abstract

Bone loss is a well-known phenomenon in the older population leading to increased bone fracture risk, morbidity, and mortality. Supplementation of eggshell membrane (ESM) is evaluated due to its possible application to prevent bone loss and usage in osteoporosis therapy. The similar organic chemical composition of ESM and human bone is described in detail as both mainly consist of collagen type I, chondroitin sulfate, dermatan sulfate, hyaluronic acid and elastan. ESM and its components are reported to improve mineralization in bone tissue. In many studies ESM intake reduced pain in patients with joint disorders and reduced inflammatory processes. Additionally, ESM improved calcium uptake in human cells. These findings in comparison with a clinical pilot study reporting pain reduction in osteoporotic patients and increased osteoblast activity in in vitro assays support ESM to be a beneficial supplement for bone health. In this systematic review we combined chemical structure analysis with clinical studies to give a more comprehensive picture with novel explanations.

## 1 Introduction

Bone loss is a well-known phenomenon in the older population leading to increased prevalence of osteoporosis (Pignolo and Ahn, 2018). Additionally, reduction in bone tissue quality according to age-related changes in bone matrix properties results in increased bone fracture risk, morbidity, and mortality (Pouresmaeili et al., 2018; Burr, 2019; Chandra and Rajawat, 2021). Therefore, osteoporosis and bone fragility fractures resulting from age-related loss in bone density are a major concern of health care for our rapidly growing and aging population.

**Fig. 1:**
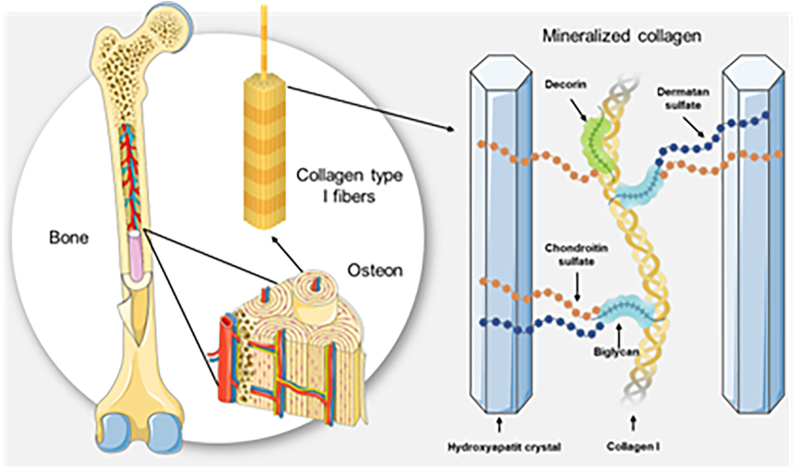
Bone composition. The role of GAGs and structure proteins for collagen type I mineralization is demonstrated.

The composition of bone tissue includes 60 % mineral phase, mainly calcium phosphate as hydroxyapatite Ca_5_[OH(PO_4_)_3_] and 40 % organic phase, including water, collagen and non-collagenous proteins (Weiner and Traub, 1992). Type I collagen accounts for 90 % by weight of the organic phase. Nevertheless, type III and V collagen can be found too in the extracellular matrix (ECM) of the human bone (Lin et al., 2020). The major part of the non-collagenous proteins are proteoglycans which are characterized as core proteins with covalently bound glycosaminoglycan (GAG) residues. The six types of GAGs found in proteoglycans include keratan sulfate, chondroitin sulfate, heparan sulfate, dermatan sulfate and hyaluronic acid (Kjellén and Lindahl, 1991). Small leucine-rich proteoglycans (SLRPs), such as biglycan, decorin, keratocan, and asporin, are important proteoglycans in the bone interacting with cell surface receptors and cytokines to regulate cellular behaviors (Kirby and Young, 2018). Additionally, decorin and biglycan are structure proteins crucial for bone density (Fig. 1).

**Fig. 2:**
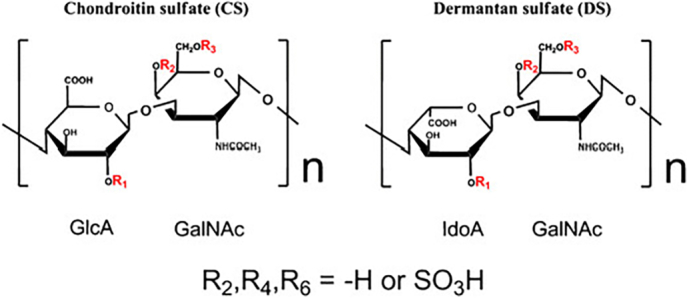
Chemical structure of chondroitin sulfate and dermatan sulfate. According to their negative charge they are able to bind mineral structures.

The covalently bound GAG residues of decorin and biglycan are chondroitin sulfate (CS) and dermatan sulfate (DS) (Hua and Jiang, 2021). Both GAGs provide very similar chemical scaffolds (Fig. 2).

Other matrix proteins which can be found in human bones are osteocalcin, playing a role in the metabolic regulation as hormone (Hauschka and Reid, 1978), and osteopontin belonging to the SIBLINGs family (Small Integrin Binding Ligand N-Glycosylated proteins). Osteopontin has been implicated as an important factor in bone remodeling. (Choi et al., 2008) Both play an important role also in avian egg shell formation but their quantity within ESM has not been determined so far (Pines et al., 1995; Fernandez et al., 2003). Studies providing evidence for osteocalcin and osteopontin as bioactive compounds in ESM are missing.

## 2 Objective

While supplementation of collagen, elastin, GAGs and other organic compounds of ECM for cartilage health is studied in detail in many clinical trials, little is known about GAGs as supplements to prevent loss of bone density during aging (Honvo et al., 2020). A natural source of GAGs and structural proteins associated with bone density is eggshell membrane (ESM). ESM mainly consists of cross-linked collagens (I, V, and X), GAGs, egg white proteins (i.e. Lysozyme) and eggshell matrix proteins (Shi et al., 2021). The aim of this systematic review is to evaluate medical appropriateness of ESM as supplement to improve bone density in elderly patients.

## 3 Comparison of bone and ESM organic chemicals

Despite the differences in protein composition (Rath et al., 2016; Lin et al., 2020), bone and ESM show many similarities in collagen proteins and GAGs (Nakano et al., 2001; Hua and Jiang, 2021). In contrast to cartilage, collagen type I is the major structural protein in bone as well as in ESM instead of collagen type II (Arias et al., 1991). Additionally, in both biomaterials CS and DS are the major residues for SLRPs. A comparison of major organic constituents of ESM and bone is given in Table 1.

**Table 1:** Comparison of organic chemical composition of bone and ESM.

Compound	Bone	ESM
Collagens	Collage type I, III, V (ratio 100:1:1)	Collage type I, V, X (ratio 100:1:1)
GAGs	Chondroitin sulfate Dermatan sulfate Hyaluronic acid	Chondroitin sulfate Dermatan sulfate Hyaluronic acid Keratan sulfate
Other proteins	Proteoglycans Elastin g-carboxyglutamic acid containing proteins Small integrin-binding ligands N-linked glycoproteins Glycoproteins	Proteoglycans Elastin Egg white proteins Eggshell matrix proteins

A comparison of the organic compounds of bone and ESM revealed that in both collagen type I is the major structure protein. Additionally, both contain high amounts of CS, DS and hyaluronic acid (HA) as part of their GAG profile. That these similarities support bone growth and bone formation finds proof in many studies investigating ESM as scaffold for bone regeneration (Torres-Mansilla et al., 2023). Comparing the organic chemistry of bone and ESM we can state the following:

I) Bone and ESM share the same structural protein collagen type I.
II) Bone and ESM have the same GAG profile. Therefore, ESM intake might improve bone formation as all basic structural elements are given.

## 4 Bioavailability of ESM products

After oral administration, GAGs like chondroitin sulfate can be transported across the small intestine in their intact form, probably by the mechanism of endocytosis (Barthe et al., 2004). In the colon and the cecum, most of the CS is absorbed in the form of the degradation products, the disaccharides. These disaccharides can be linked by glycosyltransferases, for example chondroitin synthase, after absorbtion to form chondroitin polymers (Silbert and Sugumaran, 2002). Chondroitin and dermatan polymers are sulfated in the human body by seven sulfotransferases, leading to the final structures (Mikami and Kitagawa, 2013). A biological effect following oral supplementation with GAG was demonstrated in healing of osteochondral defects in vivo. (Handl et al., 2007) Furthermore, these GAGs are the major structural elements of proteoglycans, which are essential for the water-binding capacity in connective tissues (Frantz et al., 2010) and play a crucial role in bone homeostasis (Miguez, 2020).

Digestion of oral administered collagen results in the absorption of di- and tripeptides. These collagen tripeptides provide enzymatic stability and intestinal permeability in a rat model (Sontakke et al., 2016). Further, they provide high bioactivity by stimulating matrix protein synthesis (Edgar et al., 2018). A clinical trial could proof that orally supplemented collagen peptides together with CS resulted in an improvement in collagen fiber organization in the human skin (Czajka et al., 2018). In a rat model, orally administered collagen provided beneficial effects on bone metabolism, especially in the calcium-deficient condition, without obvious undesirable effects (Wu et al., 2004).

Concerning the bioavailability of ESM products we can state the following:

I) ESM products or its derivates can at least partially be absorbed by the human intestine.
II) These products or their derivates provide biological activity after oral administration.

## 5 ESM for bone mineralization

Bone mineralization is a dynamic process of collagen assembly and mineralization including at least three independent mechanisms by which osteocytes can embed within bone matrix (Shiflett et al., 2019). Collagen type I is the major structure protein in human bones and plays a key role in bone mineralization (Bonucci, 2012). Collagen type I intake could show beneficial effects on joint and bone health (Figueres Juher and Basés Pérez, 2015). Additionally, dietary collagen hydrolysates retard estrogen deficiency-induced bone loss through blocking osteoclastic activation and enhancing osteoblastic matrix mineralization (Kim et al., 2022).

Glycosaminoglycans and proteoglycans are macromolecules of the bone and are involved in the assembly and mineralization of the extracellular matrix (Mania et al., 2009). CS constitutes about 90 % of the total GAGs in human bone (Waddington et al., 1989). Apart from CS, small amounts of DS and hyaluronic acid (HA) have been detected in human bone as part of the mineralization process (Engfeldt and Hjerpe, 1976; Malavaki et al., 2007). CS and DS are part of the PG species decorin and biglycan. Both PGs are involved in the structural organization of the bone matrix and bind collagen type I (Vynios et al., 2002). They modulate collagen fibrillogenesis, but also interact with growth factors and cytokines, such as TGF-β and BMP-2 and BMP-4, which are involved in bone homeostasis. They play a fundamental role in the regulation of bone organogenesis through the activation of receptor serine/threonine kinases and are involved in the regulation of osteoblast lineage-specific differentiation and later bone formation (Rahman et al., 2015). PGs in bone are bound to hydroxyapatite and regulate the mineralization process (Lamoureux et al., 2007). HA can promote intrafibrillar collagen mineralization by reducing the electronegativity of the collagen surface to enhance calcium ions binding capacity to create a local higher supersaturation. HA also provides additional nucleation sites and shortens the induction time of amorphous hydroxyapatite crystallization, which benefits mineralization (Wu et al., 2023).

In rat models it could be demonstrated, that orally supplemented CS alleviates diabetic osteoporosis by improving collagen mineralization (Qi et al., 2021) and that oral application of HA significantly improves bone regeneration (Li et al., 2023).

Recent studies revealed that eggshell matrix proteins, which occur in high abundance in ESM, enhance calcium transport in human cells (Daengprok et al., 2003). In a mice model it was demonstrated that intake of eggshell calcium together with ESM improves calcium absorption and deposition in comparison with eggshell calcium supplementation alone (Liu et al., 2023). Additional ESM supplementation lead to more complete, compact, and thicker trabecular structure in mice femur.

The influence of ESM supplementation on bone mineralization may be summarized as follows:

I) Collagen type I, Cs, DS and HA play important roles in bone mineralization process.
II) ESM improves calcium absorption and deposition.

## 6 Anti-inflammatory properties of ESM

Osteoarthritis (OA) is the most prevalent form of arthritis and affects cartilage and bone (Lawrence et al., 2008). Arthritic bone destruction is associated with osteoporosis and susceptibility to fragility fractures, because both phenomena reflect high inflammatory disease activity (Romas and Gillespie, 2006). The link between osteoclast, macrophage factor and pro-inflammatory cytokines, especially tumor necrosis factor-α (TNF-α) and interleukin-1 (IL-1) explain the association between inflammation and bone loss or osteoporosis (Lacativa and de Farias, 2010). Supplementation of 26 mg/kg of ESM reduced pro-inflammatory cytokines, especially (TNF-α) and (IL-1) in rats (Ruff and DeVore, 2014). These findings are supported by clinical studies proving that ESM supplementation of 300 mg/day reduced joint pain (WOMAC scale) and improved mobility (Ruff et al., 2009a; Ruff et al., 2009b; Blasco et al., 2016). In all studies ESM intake was recorded to show no adverse effects.

I) ESM reduces pro-inflammatory cytokines in patients with joint disorder.

## 7 ESM for bone density and bone formation

While eggshell calcium is well-known for its ability to prevent bone loss and osteoporosis by increasing bone density, calcium up-take and bone formation (Rovenský et al., 2003), ESM is more often used as supplement in osteoarthritis therapy. In case, a significant improvement of clinical symptoms has been reported in patients suffering from osteoarthritis (Danesch, 2014; Quintana et al., 2018). Interestingly, none of these clinical studies have associated the beneficial effect of ESM with an influence of ESM on bone formation.

A first evidence of a beneficial role of ESM concerning bone density is, that its main compounds like chondroitin sulfate play pivotal roles in bone density via retaining bound water in bone (Hua et al., 2020). Despite this evidence, only one clinical study clearly evaluated the beneficial effects of ESM on bone health in osteoporotic patients. In this study, an in-house preparation consisting of minerals like magnesium (Rondanelli et al., 2021), calcium (Flynn, 2003), phosphorus and strontium (Querido et al., 2016) known for their bone promoting activities as well as Vitamin D and eggshell membrane as main compound was used (Görögh et al., 2017). The study included fifteen elderly patients and recorded the effect of 600 mg of the preparation three times daily on pain alleviation for 20 days. The pain level of the patients treated with the given preparation considerably decreased significantly by 68 % over the treatment period of 20 days. As this effect is much stronger than that of strontium supplementation (29 %) (Boonen, 2006) and as for the other minerals included no pain reduction is reported (Morel et al., 2021), ESM is suggested to be the bioactive agent. Despite the limitations according to the modest sample size, this clinical pilot-study highlights the beneficial effects of ESM on bone health. Additionally, in the study in vitro assays were performed, providing an insight into the action mechanisms by which ESM of the in-house preparation is able to contribute to mineralized nodule formation in association with the activation of the serine/threonine kinase 1 signal transduction pathway, which is involved in the stimulation of specific gene expression required for the susceptibility of primary osteoblasts to bone formation (Raucci et al., 2008). Most interesting, in the in vitro assays the dosage of minerals and Vitamin D were adjusted to physiological concentrations. Therefore, the increased osteoblast activation seems to result from the ESM instead of the other compounds of the preparation. As a result, this study strengthens its clinical results with an explanation of the mechanism of action leading to the following key findings:

I) ESM preparations reduce pain in osteoporotic patients.
II) ESM increases osteoblast activity in vitro.

## 8 Conclusion

**Fig. 3:**
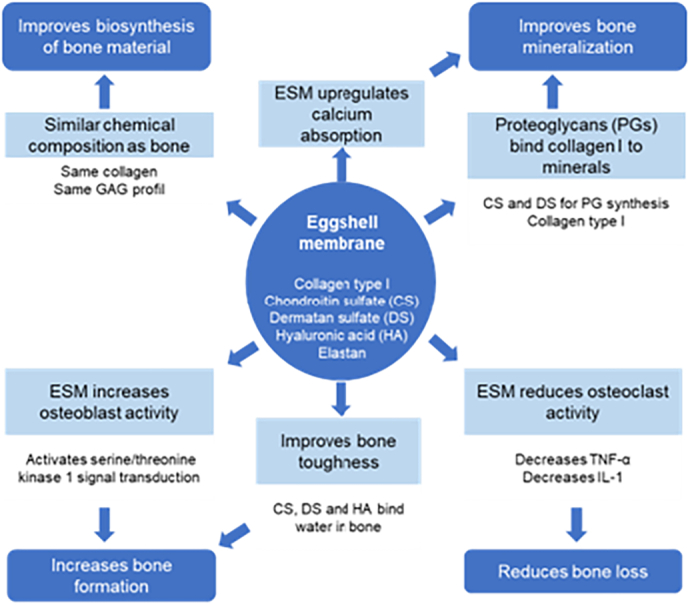
Overview of beneficial effects of ESM on bone health*.*

In this systematic review we could point out the similarities in chemical composition of collagenous and not-collagenous proteins and GAGs in bone and ESM. Single constituents as well as ESM preparations play a beneficial role in bone mineralization. ESM products can at least partially be absorbed by human intestine and they or their metabolites are bioactive after oral administration. Supplementation of ESM reduces pain in joint disorders in human and animal models and inhibits pro-inflammatory cytokines like TNF-α and IL-1. Combining these findings with the results of the study using an ESM containing in-house preparation suggests ESM as supportive and save supplementation for the prevention and therapy of patients suffering from bone loss. Of course, clinical trials with increased number of participants should be conducted in the future to give a more precise picture. Nevertheless, we can give reasonable explanations how ESM supplementation works beneficial for bone health (Fig. 3).

Major benefits of ESM supplementation to improve bone health are the improved biosynthesis of bone constituents, improved bone mineralization, decreased bone loss and increased bone formation.

## Funding

Open access funding provided by University of Graz. Karl-Franzens-Universität Graz.

## Human and animal rights and informed consent

This article does not contain any studies with human or animal subjects performed by any of the authors.

## Role of the funder/sponsor

The funders had no role in the design of the study.

## CRediT authorship contribution statement

**Johannes-Paul Fladerer:** Writing – review & editing, Writing – original draft, Supervision, Project administration, Methodology, Investigation, Funding acquisition, Formal analysis, Conceptualization. **Selina Grollitsch:** Writing – review & editing, Visualization, Validation, Software, Resources, Formal analysis, Data curation.

## Declaration of competing interest

JPF reported receiving personal fees from Apomedica Pharmazeutische Produkte GmbH as employee.The authors have no conflict of interest to declare.

## Data Availability

Data will be made available on request.
